# The relationship between visual health and influencing factors among primary and secondary school students: a survey based in the Ningxia Hui Autonomous Region of China

**DOI:** 10.3389/fmed.2024.1457465

**Published:** 2024-10-03

**Authors:** Jiyan Xu, Mengjiao Sang, Weiwei Xu, Kaijie Feng

**Affiliations:** ^1^Department of Recreation and Social Sports, Capital University of Physical Education and Sports, Beijing, China; ^2^School of Pharmaceutical Sciences, Shandong University, Jinan, China; ^3^College of Humanities and Law, Beijing University of Chemical Technology, Beijing, China; ^4^Department of Physical Education and Research, Peking University, Beijing, China

**Keywords:** myopia, visual health, lifestyle behaviors, physical activity level, gender, masking effect, moderating effect

## Abstract

**Introduction:**

The myopia rate of young people around the world, especially in China, has continued to rise, and the vision health of primary and secondary school students has gradually become a global concern. It was to explore the current characteristics of vision status and their intrinsic links to influencing factors of the Ningxia Hui Autonomous Region primary and secondary school students under regional characteristics.

**Methods:**

1,670 primary and secondary school students in Ningxia Hui Autonomous Region were surveyed using Vision examination, International Physical Activity Scale, Lifestyle Behavior Scale. The data were analyzed using SPSS 25.0 and AMOS 23.0.

**Results:**

The prevalence of myopia among primary and secondary school students in the Ningxia Hui Autonomous Region was 27.3%, with rates of 31.5% for girls and 23.4% for boys. There was a significant negative correlation between lifestyle behaviors (*r* = -0.36, *p* < 0.01) and physical activity level (*r* = –0.06, *p* < 0.05) with vision status. The physical activity level played a significant masking effect between lifestyle behaviors and vision status (a*b = 0.002, c’ = –0.044). Gender moderated the effects of lifestyle behaviors on physical activity level and vision status, as well as the effects of physical activity level on vision status. These factors constituted a mixed model with mediating and moderation. The model fitted well (RMSEA = 0.028, CFI = 0.951, NFI = 0.918).

**Discussion:**

The prevalence of myopia among primary and secondary school students in this region is lower than the national average in China, and it is associated with lifestyle behaviors, physical activity level, and gender. When effectively controlled, the physical activity level significantly will reduce the prevalence of myopia as a masking variable. Gender, as a moderating variable, provides theoretical support for the classification and prevention of myopia. Regional surveys enriched the global database on adolescent myopia research, revealing the characteristics and common factors of visual problems in adolescents. Relevant departments and schools should integrate eye care into the national health security system, implement policies related to myopia prevention and control and safeguard the visual health of primary and secondary school students.

## 1 Introduction

In recent years, the global prevalence of myopia among adolescents has been steadily increasing, making the visual health issues of primary and secondary school students a focus of global attention. In October 2019, the World Health Organization released the “World Report on Vision,” indicating that at least 2.6 billion people worldwide suffered from visual impairment or blindness, with myopia becoming one of the most prevalent eye disorders. The report also highlighted the alarming situation of myopia prevalence among Chinese adolescents (1). According to the latest data released by the National Health Commission of China in 2023, the overall myopia rate among Chinese children and adolescents was 52.7%. Specifically, the myopia rates among primary school students, junior high school students, and senior high school students are 35.6, 71.1, and 80.5% respectively, showing a trend toward younger age groups. Visual impairment can have adverse effects on the physical and mental development, quality of life, and learning outcomes of children and adolescents, while also increasing the risk of high myopia and related vision disorders in adulthood (2).

The visual condition is one of the important indicators reflecting the physical health level of children and adolescents. There are many influencing factors for adolescent vision, and the related theories and mechanisms are quite complex. Among them, lifestyle behaviors and physical activity levels have certain impacts on the visual health of adolescents (3). The lifestyle behaviors of children and adolescents may be the main reason for the rapid increase in myopia rates, with factors such as sports and reading time contributing to myopia (4–6). The lifestyle behaviors investigated in this study included four sub-behaviors: sleeping behavior, eating behavior, sports behavior, and eye behavior. Research has shown that there is a negative correlation between children’s physical activity levels and myopia (7). Subsequent studies had further explored the relationship between physical activity levels and myopia (8, 9). Physical activity for the purposes of this study was defined as any physical activity that elicits skeletal muscle contraction and causes an increase in energy expenditure on top of resting energy expenditure (10). Gender may also be an important factor influencing the health of children and adolescents, as myopia, physical activity levels, and lifestyle behaviors exhibit different characteristics between boys and girls (11). However, the specific relationships require further verification, and the mechanisms remain unclear.

Currently, research on the visual health of primary and secondary school students often focused on the analysis of individual influencing factors. However, this study aims to explore the relationship between the prevalence of myopia among primary and secondary school students and various factors such as lifestyle behaviors and physical activity levels under regional characteristics using the Ningxia Hui Autonomous Region as an example.

Based on the above analysis, this study proposed four research hypotheses: (H1) The lifestyle behaviors of primary and secondary school students have a negative impact on their visual status; (H2) The level of physical activity among primary and secondary school students are negatively correlated with their visual status; (H3) Physical activity levels mediate the relationship between lifestyle behaviors and visual status; (H4) Gender moderates the relationship between lifestyle behaviors, physical activity levels, and visual status. Mediating can elucidate how lifestyle behaviors influence myopia, while moderation can reveal the extent to which lifestyle behaviors affect physical activity levels and myopia. Building upon a precise understanding of the visual status and influencing factors among students in this region, this study aims to provide guidance for myopia prevention and control efforts in the area, thus contributing to the global theoretical framework and practical experience in myopia prevention and control among adolescents.

## 2 Method

### 2.1 Research subjects

The research subjects of this study are primary and secondary school students in the Ningxia Hui Autonomous Region of China, ranging in age from 6 to 16 years old. The ages of the students are 6–13 years old at the primary level and 13–16 years old at the secondary level. Ningxia Hui Autonomous Region, located in the northwest of China, is an area inhabited by multiple ethnic groups, characterized by unique geographical and cultural features. In comparison to other developed cities, this region exhibits certain differences in educational resources, economic conditions, and other aspects. Additionally, its diverse lifestyle habits and physical activities may have an impact on the visual health of primary and secondary school students. Focusing on this region for investigation can make research on visual health issues among primary and secondary school students more characteristic, aiding in the deeper understanding of the commonalities and specificities of visual health problems. A questionnaire survey was conducted on these students from September to October 2023 using random sampling. A total of 1,670 valid questionnaires were collected for this study. Among them, there were 840 male students, accounting for 50.3%, and 830 female students, accounting for 49.7%. Primary school students accounted for 1,360, making up 81.4%, while secondary school students accounted for 310, making up 18.6%.

### 2.2 Research method

#### 2.2.1 Vision examination

The research team for this study included at least one ophthalmologist licensed as a national practitioner in ophthalmology, with expertise in optometry, and several professionals holding certifications as technicians or nurses in vision-related fields. Two types of tests were used for the visual acuity assessment. One is the use of a standard logarithmic visual acuity meter in accordance with the national standard (GB 11533), which is placed 5 m in front of the examined eye, and before the test, the requirements and purposes are made clear, and the subject is asked to speak or make the corresponding gestures to indicate the direction of the visual standard gap (12). Secondly, a computerized optometry instrument conforming to the pharmaceutical industry standard (YY 0673-2008) was used, and under the condition of non-ciliary muscle paralysis, each eye of the examinee was measured three times and the average value was taken (13). The criteria for diagnosing myopia were based on the following standard: uncorrected visual acuity <5.0 and equivalent spherical power under non-cycloplegic conditions < −0.50 diopters cylinder. Prevalent conditions such as strabismus, pathological high myopia and retinal disease were excluded prior to the study.

#### 2.2.2 International physical activity questionnaire-short form

The International Physical Activity Questionnaire-Short Form (IPAQ-SF) consists of 7 items. The first 6 items mainly assess the physical activity of the subjects, while the 7th item evaluates the sitting time of the subjects. Walking, moderate-intensity activity, and high-intensity activity are assigned values of 3.5, 4.5, and 8.0, respectively. Following previous experiences, outliers were excluded and data were organized. Physical activity was categorized into low, moderate, and high groups. The criteria for the high group were as follows: (1) At least 3 days of high-intensity activity per week, with a total weekly physical activity level of at least 1,500 MET*minutes/week; (2) At least 7 days of a combination of three intensities per week, with a total weekly physical activity level of at least 3,000 MET*minutes/week. The criteria for the moderate group were as follows: (1) At least 3 days of high-intensity activity per week, with an average of at least 20 min per day; (2) At least 5 days of moderate-intensity activity per week, with an average of at least 30 min per day; (3) At least 5 days of a combination of three intensities per week, with a total weekly physical activity level of at least 600 MET*minutes/week. The criteria for the low group were as follows: (1) No physical activity; (2) Engaging in physical activity but not meeting the criteria for the moderate or high group (meeting any one of the grouping criteria is considered to meet the criteria for that group). In this study, the variable of physical activity grouping was used as the assessment indicator for physical activity level.

#### 2.2.3 Lifestyle behaviors scale

The Behavioral Assessment Scale is composed of multiple scales, including sleep behavior, dietary behavior, eye use behavior, and exercise behavior, forming four dimensions with a total of 18 items. Each dimension consists of 4, 3, 8, and 3 items, respectively. There are two types of questions: one uses a dichotomous test (“yes” scores 1 point, “no” scores 0 points), and the other uses the Likert scoring method, with options of 3 and 5, scoring positively from “1 to 3” and “1 to 5” respectively. The theoretical total score of the scale is 58 points, and quantitative data were used for analysis in this study. After the formation of the scale, content validity was judged by experts, and after meeting the requirements, the scale’s structural validity and reliability were tested. After the first round of distribution and collection, 144 responses were obtained. The exploratory factor analysis confirmed the structural validity, and then the second round of distribution (*n* = 1,670) was conducted.

### 2.3 Method of administration and data processing

The study collected data through online questionnaire completion from September 28 to October 12, 2023, using the Questionnaire Star online survey platform. Participants completed the questionnaire based on informed consent and voluntary participation. Demographic information such as gender (male = 1, female = 0), age, and grade level (primary school = 0, junior high school = 1) was gathered during the survey. After questionnaire retrieval, data were processed quantitatively using Excel. Descriptive statistics, correlation analysis, regression analysis, and moderation effect testing were conducted using SPSS 25.0 and SPSSAU. Mediating effects were tested using Bootstrap estimation, and the hypothetical model was examined using AMOS 23.0.

## 3 Results and analysis

### 3.1 Results of questionnaire reliability and validity test

The distribution of sample characteristic values of International Physical Activity Questionnaire-Short Form obtained in this measurement was 1.55 ± 0.79 (*M ± SD*), with *Cronbach*’s *α* of 0.791. The data obtained in Lifestyle behaviors Scale were analyzed through exploratory and confirmatory factor analyses, with the results shown in Table 1, where all items met the requirements. Skewness values of the items ranged from 0.453 to 1.367, kurtosis values ranged from 0.095 to 1.624, and the minimum standard deviation was 0.774, with K-S normality test (*p* < 0.001). The scale’s *Cronbach’s α* was 0.686, split-half reliability was 0.727, and item-total correlations ranged from 0.289 to 0.514 (*p* < 0.01, as shown in Table 1). The reliability coefficient of the scale falls between 0.6 and 0.7, which is considered acceptable.

**Table 1:** Exploratory factor analysis and validation factor analysis index of lifestyle behavior scale.

Scale	Exploratory factor analysis				Validation factor analysis							
	KMO	Bartlett-test	df	*p*	χ^2^/df	GFI	NFI	IFI	NNFI	CFI	RMSEA	SRMR
Lifestyle behaviors	0.709	5095.411	153	<0.001	7.093	0.955	0.903	0.914	0.907	0.912	0.054	0.046

### 3.2 Common methods bias test

The results of the Harman’s one-factor test for common bias showed that the eight factors explained 60.28% of the variation in total, with the first factor explaining 30.96% of the variation. This indicates that there are no significant common methods bias among the variables and the research can be continued (14).

### 3.3 Descriptive statistics, tests of variance and correlation analysis

As shown in Table 2, the independent sample *t*-test and *χ*^2^ test analysis found that there were significant gender differences in myopia (*χ*^2^ = 13.823, *p* < 0.001) and physical activity level (*t* = −3.079, *p* < 0.001), while there was no significant gender difference in lifestyle behavior (*t* = 0.065, *p* = 0.130 > 0.05). Among them, the myopia rate of boys was 23.4%, which was lower than that of girls at 31.5%. The physical activity level of boys ( $\bar{x}$ ± *s* = 1.61 ± 0.84) ( $\bar{x}$ ± *s* = 1.61 ± 0.84) was higher than that of girls ( $\bar{x}$ ± *s* = 1.49 ± 0.74). However, in lifestyle behavior, there was little difference between boys ( $\bar{x}$ ± *s* = 47.01 ± 3.77) and girls ( $\bar{x}$ ± *s* = 47.03 ± 3.87). Among the four sub-behaviors of life behavior, only sports behavior showed significant gender differences (*t* = −4.057, *p* < 0.001), and boys’ sports behavior ( $\bar{x}$ ± *s =* 4.59 ± 0.68) was better than girls’ ( $\bar{x}$ ± *s =* 4.45 ± 0.79). However, there were no significant gender difference in sleep behavior (*t* = −0.538, *p* = 0.213 > 0.05), eating behavior (*t* = 0.640, *p* = 0.251 > 0.05), and eye behavior (*t* = −0.206, *p =* 0.645 > 0.05).

**Table 2:** Descriptive statistics and gender difference of myopia, lifestyle behaviors and physical activity level.

Category	Myopia		Physical activity level	Lifestyle behaviors	Lifestyle behaviors			
	Yes	No			Sleep behavior	Eating behavior	Sports behavior	Eye behavior
Male (*n* = 840)	23.4%	76.6%	1.61 ± 0.84	47.01 ± 3.77	8.07 ± 1.27	9.44 ± 1.90	4.59 ± 0.68	25.05 ± 2.57
Female (*n* = 830)	31.5%	68.5%	1.49 ± 0.74	47.03 ± 3.87	8.03 ± 1.32	9.50 ± 1.98	4.45 ± 0.79	25.02 ± 2.57
*t*/χ^2^	13.823		−3.079	0.065	−0.538	0.640	−4.057	−0.206
*p*	<0.001		<0.001	0.130	0.213	0.251	<0.001	0.645
Cohen’s d	−0.179		0.151	−0.005	0.031	−0.031	0.189	0.011

The correlation analysis results are shown in Table 3. Both lifestyle behaviors (*r* = −0.36, *p* < 0.01) and physical activity level (*r* = −0.06, *p* < 0.05) had significant negative correlations with myopia, which indicates that as the subjects’ life behavior scores and physical activity levels increased, their myopia rates decreased instead. Additionally, there was a significant negative correlation between physical activity level and lifestyle behaviors (*r* = −0.13, *p* < 0.01). Since there were significant correlation relationships among all pairs of variables, further regression analysis can be conducted.

**Table 3:** Correlation analysis.

Variable	*M ± SD*	1	2	3
1. Myopia	0.27 ± 0.45	1		
2. Lifestyle behaviors	47.02 ± 3.82	−0.36**	1	
3. Physical activity level	1.55 ± 0.79	−0.06*	−0.13**	1

M, mean; SD, standard deviation; **p < 0.01, *p < 0.05.

### 3.4 Mediating effect test

Mediating role refers to the study of the effect of the independent variable on the dependent variable, whether it will affect the dependent variable through the mediating variable. Mediating effect test were conducted using SPSS AU. After the raw data were imported, the independent, dependent, and mediating variables were imported into the appropriate locations. Using the Bootstrap estimation method, a random repeated sampling was conducted on a sample of 1,670 primary and secondary school students to estimate the 95% confidence interval of Mediating effects. The results are shown in Table 4. According to the judgment method of Mediating effect and masking effect proposed by Wen and Ye (15), the indirect effect value of physical activity level on the impact of lifestyle behaviors on myopia in this study is 0.002, and the 95% confidence interval of the estimate is [0.006, 0.021]. In this case, both coefficients a and b are significant, and the Mediating effect value (0.002) and the direct effect value (−0.044) have opposite signs, indicating that the physical activity level of primary and secondary school students plays a masking effect in the process of the impact of lifestyle behaviors on myopia.

**Table 4:** Result of mediating effect test.

Term	Total effect	*a*	*b*	Intermediary effect	*SE*	*z*	*p*	a*b (95% *BootCI*)	Direct effect	Inspection conclusion
Lifestyle behaviors → Physical activity level → Myopia	−0.042**	−0.026**	−0.059**	0.002	0.004	0.403	0.006	0.006~0.021	−0.044**	Masking effect

### 3.5 Moderating effect test

According to Wen Zhonglin’s three-step test for mediating with moderation (15), the variables were gradually centralized and regressed. The results are shown in Table 5. Models 1–2 used physical activity level as the dependent variable, while models 3–6 used myopia as the dependent variable. In model 2 lifestyle behavior (*B* = −0.016, *p* < 0.05), gender (*B* = 1.040, *p* < 0.05), and the interaction between lifestyle behaviors and gender (*B* = −0.020, *p* < 0.05) all had significant effects on physical activity level, indicating that gender played a modulating role in the process of lifestyle behavior affecting myopia. Model 3 showed the correlation coefficients of the variables when physical activity level plays a mediating role in the process of lifestyle behaviors affecting myopia. After adding the gender variable, correlation coefficients for life behavior (*B* = −0.253, *p* < 0.01), physical activity level (*B* = −0.368, *p* < 0.01), and gender (*B* = −0.416, *p* < 0.05) were significant. Based on this, the interaction terms of lifestyle behaviors and gender, as well as physical activity level and gender, were added in sequence. Correlation coefficients were significant for lifestyle behaviors (*B* = −0.283, *p* < 0.01), physical activity level (*B* = −0.219, *p* < 0.01), gender (*B* = −2.677, *p* < 0.05), UX (*B* = 0.058, *p* < 0.05) and UW (*B* = −0.279, *p* < 0.05), indicating that gender played a modulating role in the process of both lifestyle behaviors and physical activity level affecting myopia.

**Table 5:** Results of regression analysis.

	Physical activity level		Myopia			
	Model 1	Model 2	Model 3	Model 4	Model 5	Model 6
Constant	2.768**	2.255**	11.380**	11.534**	13.208**	12.686**
Lifestyle behaviors (X)	−0.026**	−0.016*	−0.253**	−0.253**	−0.290**	−0.283**
Physical activity level (W)			−0.389**	−0.368**	−0.362**	−0.219**
Gender (U)		1.040*		−0.416**	−3.803**	−2.677*
UX		−0.020*			0.074*	0.058*
UW						−0.279*
*R* ^2^	0.016	0.023	0.140	0.144	0.146	0.148
*ΔR* ^2^	0.015	0.022	0.139	0.209	0.212	0.214

**Figure 1:**
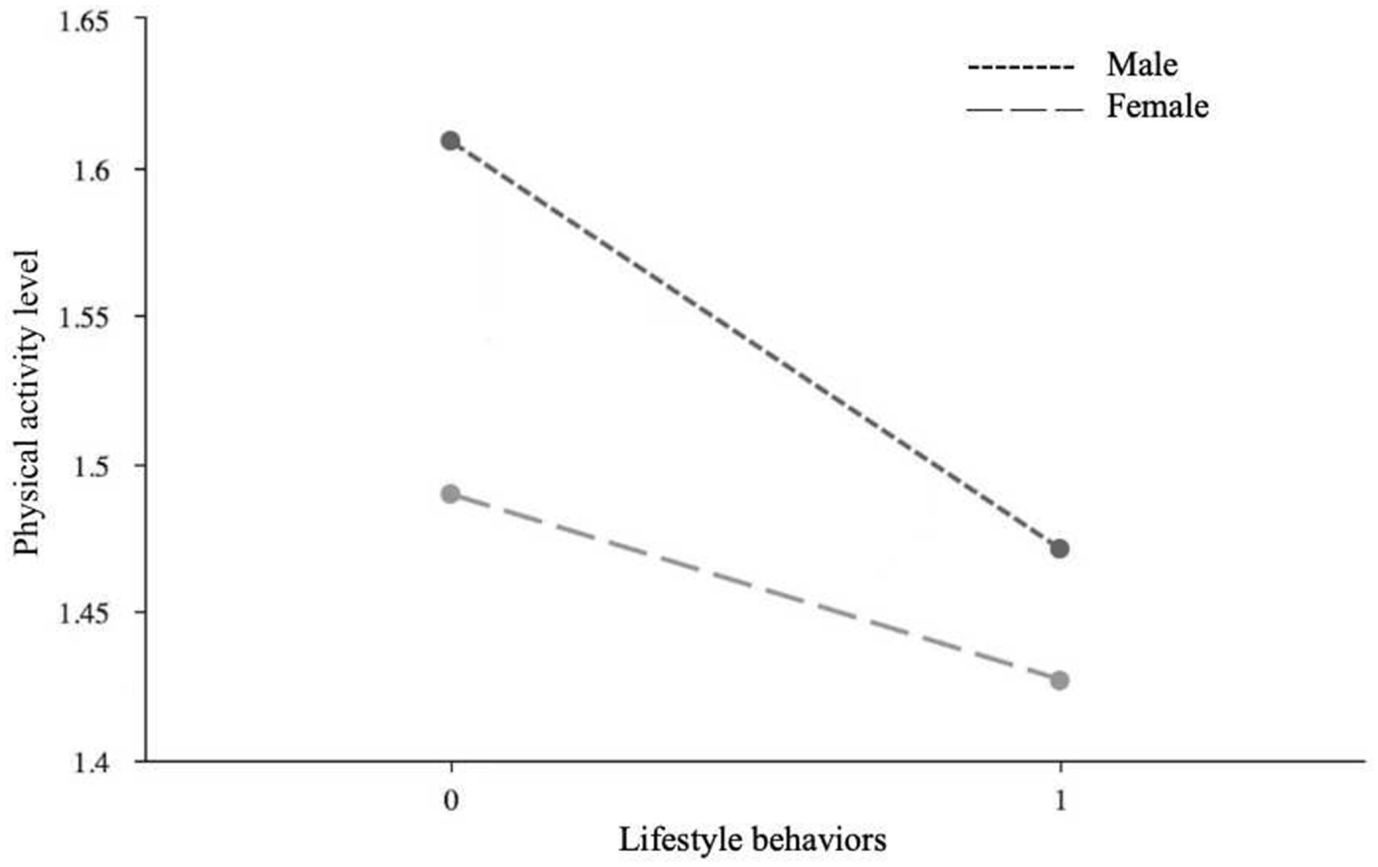
The moderating role of gender between lifestyle behaviors and physical activity level.

**Figure 2:**
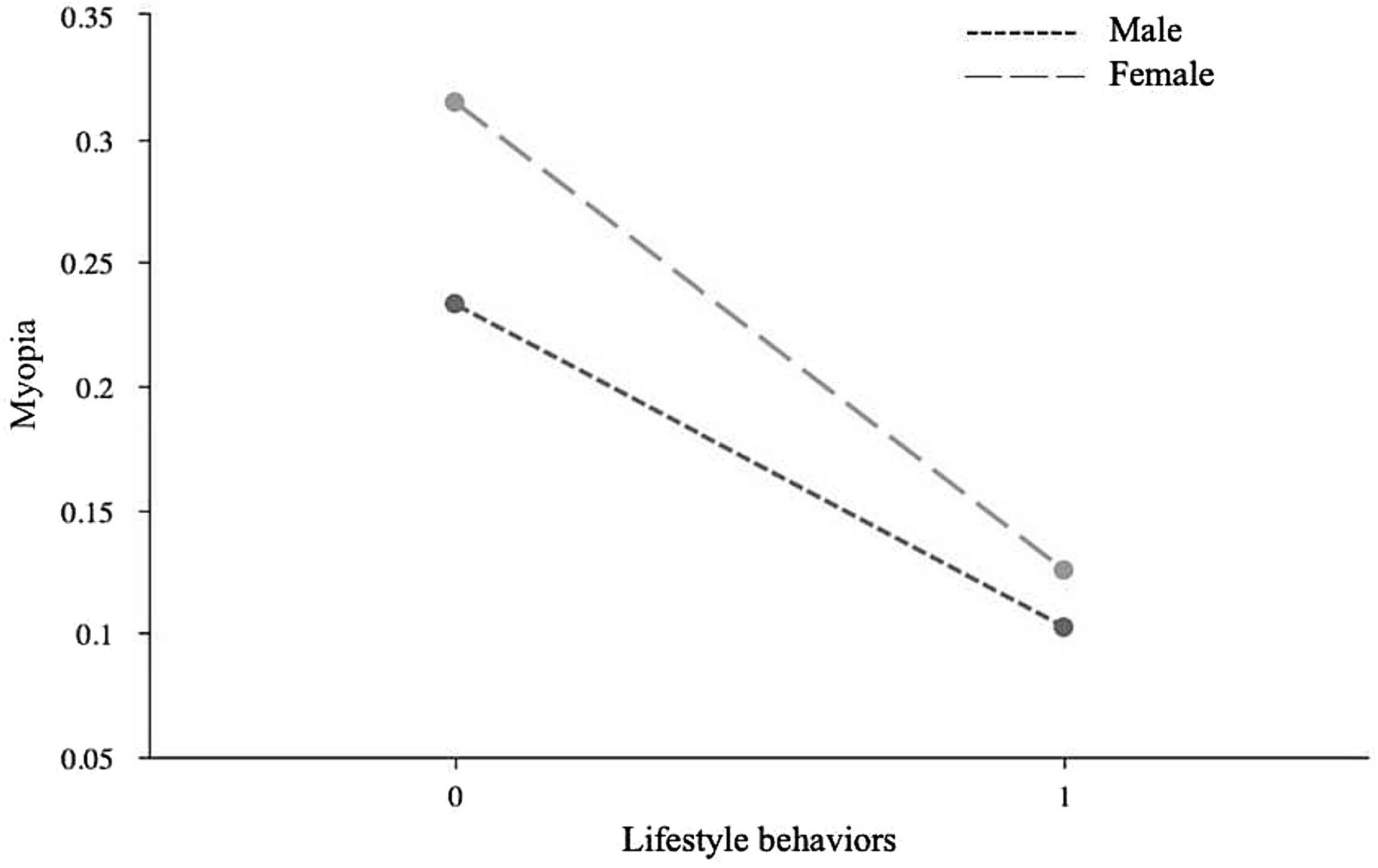
The moderating role of gender in the association between lifestyle behaviors and myopia.

**Figure 3:**
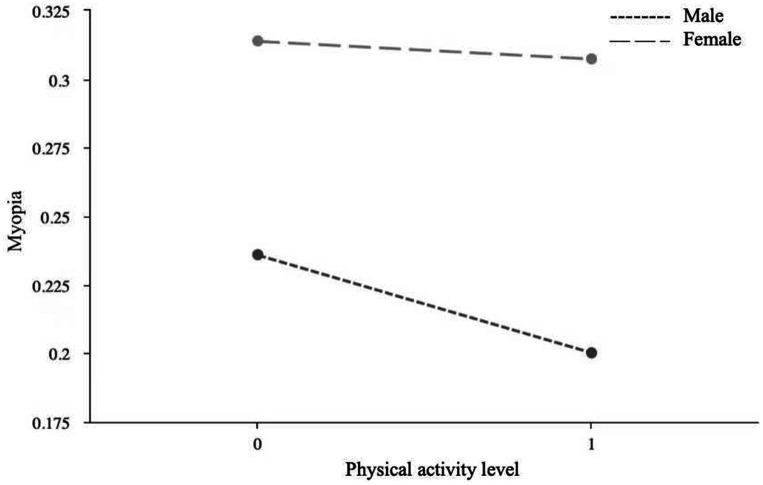
The moderating role of gender in the association between physical activity level and myopia.

Due to the significant moderating role of gender, a simple slope analysis was conducted to investigate the moderating effects of gender on the relationships between lifestyle behaviors and myopia, between lifestyle behaviors and physical activity level, and between physical activity level and myopia. First, a simple effect analysis diagram was drawn to illustrate the impact of lifestyle behaviors on physical activity level, as shown in Figure 1. The results indicated that the effect of female lifestyle behaviors on physical activity level (*B* = −0.06, *p* < 0.05) was lower than that of males (*B* = −0.14, *p* < 0.01), suggesting that changes in male lifestyle behaviors have a more significant impact on physical activity level than females. Second, a simple effect analysis diagram was plotted to show the impact of lifestyle behaviors on myopia, as depicted in Figure 2. It revealed that the effect of male lifestyle behaviors on myopia (*B* = −0.13, *p* < 0.01) was lower than that of females (*B* = −0.19, *p* < 0.01), indicating that changes in female lifestyle behaviors have a more significant impact on myopia than males. Third, another simple effect analysis diagram was drawn to demonstrate the impact of physical activity level on myopia, as shown in Figure 3. The results showed that the effect of male physical activity level on myopia was significant (*B* = −0.01, *p* < 0.05), while the effect of female physical activity level on myopia was not significant (*B* = −0.04, *p* > 0.05).

### 3.6 Model testing with mediating and moderating

**Figure 4:**
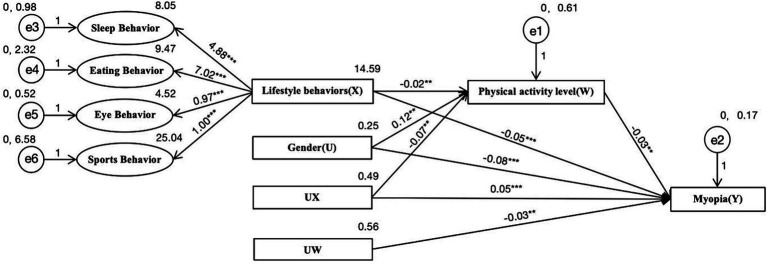
Hybrid models of moderated and mediated. * indicates *p* < 0.05, ** indicates *p* < 0.01, and *** indicates *p* < 0.001.

Using AMOS 23.0 software, the study constructed a model to analyze the relationship between lifestyle behaviors, physical activity levels, gender, and myopia, and analyzed the impact pathways. Since the chi-square value increases with the sample size, causing any model to be rejected, the study did not use the chi-square value as a criterion for judging the goodness of fit of the model. As shown in Figure 4, the absolute values of the Critical Ratio (CR) values for all impact pathways in the model were greater than 1.96, indicating that the parameter significance exceeded the 95% confidence level, and therefore the null hypothesis was not rejected. Generally, smaller SE values indicate more accurate parameter estimation, and all SE values for the paths in the model were less than 1, indicating that the parameter estimates were quite accurate. Furthermore, all impact pathways met the statistical significance standard (*p* < 0.05). When evaluating the model, multiple fit indices were typically used for testing. The study suggested that the statistical results must report the CFI and RMSEA indices (16). This model’s RMSEA was 0.028, which is less than 0.05 (STEIGER and LIND believe that an RMSEA value <0.08 is acceptable, while <0.05 indicates good fit), and the CFI was 0.951, which is greater than 0.95. In addition, the NFI was 0.918, which is greater than 0.9, indicating a good model fit. The model established revealed that the influence of lifestyle behaviors on visual acuity is controlled by physical activity levels and gender. Improvements in lifestyle behaviors and physical activity level can effectively reduce the incidence of myopia, and gender was used as a categorical variable to provide a basis for categorizing the implementation of myopia prevention and control efforts.

## 4 Analysis and discussion

This study utilized visual acuity examinations, the IPAQ-SF, and the Lifestyle Behavior Scale to investigate the visual status and influencing factors among 1,670 primary and secondary school students of the Hui ethnic group in the Ningxia Hui Autonomous Region. The study explored the mechanisms underlying the relationship between lifestyle behaviors and visual status. The results indicated that the prevalence of myopia among primary and secondary school students was 27.3%, lower than the national average. This may be attributed to the region’s emphasis on visual health education and active efforts toward adolescent myopia prevention and control. The prevalence of myopia was higher among females (31.5%) than males (23.4%), and higher among secondary school students (49.7%) than primary school students (22.3%). Negative lifestyle behaviors were found to predict myopia, with physical activity levels playing a masking role, and gender serving as a moderator. The following sections will delve into the relationship and mechanisms among lifestyle behaviors, physical activity levels, gender, and visual status.

Lifestyle behaviors, including sleep, diet, physical activity, and eye usage, were found to be significantly negatively correlated with visual status. Prolonged use of electronic devices and extended reading or writing times increase eye usage, leading to visual fatigue (17). The prevalence of myopia among those experiencing visual fatigue is notably higher than in the normal population (18). Research has shown that sleep duration is one of the influencing factors for myopia, with sleep duration being negatively correlated with the prevalence of myopia (19–21). Malnutrition is also a significant factor contributing to myopia. Regular dietary habits and a balanced nutritional intake are beneficial for the prevention of myopia (22). In this study, the mean scores for lifestyle behaviors and sub-behaviors, including sleep, diet, physical activity, and eye usage, among the group with impaired vision were 44.78, 7.66, 8.23, 4.27, and 24.21, respectively. These scores were lower than those of the group with normal vision, which were 47.86, 8.20, 9.71, 4.62, and 25.35, indicating that the lifestyle behaviors and sub-behaviors of the group with impaired vision were poorer than those of the group with normal vision.

Physical activity levels were found to be significantly negatively correlated with lifestyle behaviors and myopia. In the process of lifestyle behaviors affecting visual status, physical activity levels acted as a masking variable, enhancing the impact of lifestyle behaviors on myopia. Physical exercise can effectively protect the visual health of children and adolescents (23). Moderate-intensity physical activity significantly reduces the risk of myopia, while decreased physical activity levels can lead to visual impairment (24, 25). Participation in physical activities can effectively prevent the occurrence of myopia and to some extent improve the visual status of those already myopic (26). Research has shown that higher levels of physical activity and leisure activities are associated with better hyperopia diopter and lower myopia rates (27). Intraocular pressure refers to the pressure of the contents of the eyeball on the inner wall of the eye. The intraocular pressure of the group with impaired vision was higher than that of the normal group (28). Wang et al. pointed out through experiments that moderate to high-intensity exercise can effectively reduce intraocular pressure, while low-intensity exercise has little effect on intraocular pressure (29). In this study, 68% of students in the group with impaired vision engaged in low-intensity physical activity. Among those engaging in low, moderate, and high-intensity physical activity, 29.1, 26.6, and 22.3% of students were myopic, respectively. This indicates that the level of physical activity in the group with impaired vision was lower than that of the normal group, and as physical activity levels increased, the myopia rate among students decreased.

Gender differences in the field of health have been studied extensively, with researchers conducting numerous studies on the relationship between gender and individual physical and mental health (30, 31). Tong et al. found through analysis that the prevalence of myopia is higher among females than males, and there are several reasons for this phenomenon (32). Firstly, males tend to engage in physical exercise more than females, possibly due to differences in physiological structure and psychological needs between genders (33). In this study, 37.9% of male students engaged in moderate to high-intensity physical activities, compared to only 33.9% of female students, with the mean physical activity level of males being 1.61, which is higher than that of females at 1.49. Secondly, there are differences in lifestyle habits between males and females, with females more likely to exhibit adverse eye usage behaviors than males (34). Further analysis revealed that both males and female’s lifestyle behaviors have predictive effects on visual status. Males can prevent the occurrence of myopia by increasing their level of physical activity, while females can mitigate the risk of myopia by improving lifestyle behaviors and increasing participation in sports activities.

Research has shown that the sensitive period for visual impairment generally falls between 6 months and around 14 years old, with school-aged children being in a crucial stage (35). Addressing vision problems in adolescents should focus on both prevention and treatment, with prevention being more crucial. Therefore, emphasis should be placed on preventing myopia in adolescents by implementing regular screening mechanisms, improving their lifestyle behaviors, and encouraging their active participation in physical activities. Given that school-aged children are in a critical stage of physical and mental development, educational measures should be taken cautiously, ensuring equal treatment of boys’ and girls’ participation in sports. On one hand, relevant authorities should integrate eye care into the national health security system, encourage high-quality ophthalmic research, apply research findings to prevention and control efforts, promote eye care knowledge, and enhance public awareness of eye health. On the other hand, a collaborative approach between families, schools, and communities should be adopted. At the family level, parents should properly address gender differences, actively encourage students to participate in sports, and instill in them a correct understanding of physical education. At the school level, efforts should be made to prioritize health education, increase attention to adolescent vision, and ensure comprehensive physical education classes. Additionally, efforts should focus on incorporating myopia prevention and control into the region’s school health education, improving myopia monitoring and prevention mechanisms. Educators should change the situation of evaluating students based on scores alone and actively guide students to participate in physical activities. Physical education, as a crucial means to improve health and reduce myopia rates among adolescents, should be highly valued for its role in adolescent health. Physical education teachers should adhere to the requirements of the “Compulsory Education Physical Education and Health Curriculum Standards (2022 Edition),” teach students health knowledge, cultivate their physical abilities, healthy behaviors, and sports ethics. Attention should also be paid to the physiological and psychological differences between boys and girls, selecting sports activities that align with their physical and mental development, and treating their sports participation equally. At the community level, efforts should be made to actively promote myopia awareness, organize eye care activities, enhance adolescents’ awareness of eye health, and collaborate with ophthalmic institutions to conduct vision checks and monitoring in communities. Relevant departments and primary and secondary schools should integrate eye care into the national health security system, implement policies related to myopia prevention and control, and effectively safeguard the visual health of primary and secondary school students.

## 5 Conclusion

The myopia prevalence among primary and secondary school students in the Ningxia Hui Autonomous Region is lower than the national average for Chinese students. The occurrence of myopia is correlated with lifestyle behaviors, physical activity levels, and gender. The model established revealed that the influence of lifestyle behaviors on visual acuity is controlled by physical activity levels and gender. Improving lifestyle behaviors can effectively reduce the incidence of myopia. When physical activity levels are controlled as a confounding variable, the incidence of myopia decreases significantly. Gender, acting as a moderating variable, provides theoretical support for the classification-based myopia prevention and control efforts. Regional surveys have enriched the global database on adolescent myopia research, revealing the characteristics and common factors of adolescent vision problems, thus enriching existing theories on the causes of myopia and providing a theoretical basis for better implementation of youth myopia prevention and control.

## 6 Research limitations and outlook

Firstly, the study was constrained by time and budget limitations, leading to the selection of a region with distinct ethnic characteristics for the survey. While the results may lack broader applicability due to the limited scope, they still hold practical significance for the area under investigation.

Secondly, the occurrence and influencing factors of myopia are complex and multifaceted. Future research within ethnically specific populations could further refine lifestyle factors and utilize artificial intelligence techniques such as big data to investigate the causes and mechanisms of myopia occurrence, thereby providing more precise recommendations for myopia prevention and control.

Thirdly, future research could involve comparisons with other regions to explore the causes of differences in myopia prevalence rates across different areas, thus enhancing and refining the database for myopia prevention and control efforts.

## Data Availability

The raw data supporting the conclusions of this article will be made available by the authors, without undue reservation.
